# P-1545. Impact of Perinatal Exposure and Colonization by Drug-resistant Enterobacterales on Infant Gut Microbiota

**DOI:** 10.1093/ofid/ofaf695.1725

**Published:** 2026-01-11

**Authors:** Leena B Mithal, Weitao Shuai, Noelle Samia, Abigail Aron, Alima Sajwani, Aspen Kremer, Sebastian Otero, Erica Hartmann, Mehreen Arshad

**Affiliations:** Ann and Robert H. Lurie Children's Hospital of Chicago, Chicago, Illinois; Northwestern University, Evanston, Illinois; Northwestern University, Evanston, Illinois; Anne & Robert H. Lurie Children’s Hospital of Chicago, Chicago, Illinois; Ann & Robert H. Lurie Children's Hospital, Chicago, Illinois; Ann and Robert H. Lurie Children’s Hospital, Chicago, Illinois; University of Chicago, Chicago, Illinois; Northwestern University, Evanston, Illinois; Lurie Children's/Northwestern, Chicago, Illinois

## Abstract

**Background:**

Antibiotic-resistant Enterobacterales, including beta-lactamase producing (BL-E) and ceftriaxone resistant (CefR-E) strains, are prevalent in the U.S. and globally. Our objective was to assess the impact of resistant Enterobacterales exposure and perinatal colonization on infant microbiota development in healthy parent-infant dyads.

**Table 1.**
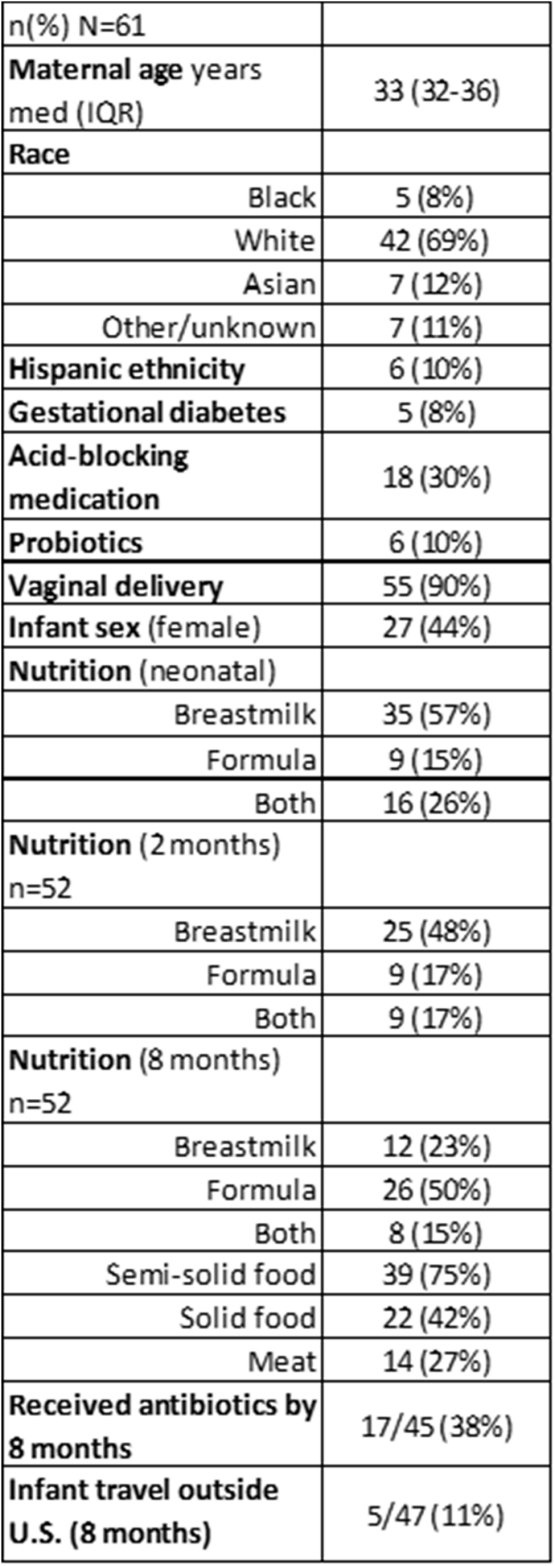
Demographics and clinical data

**Figure 1.**
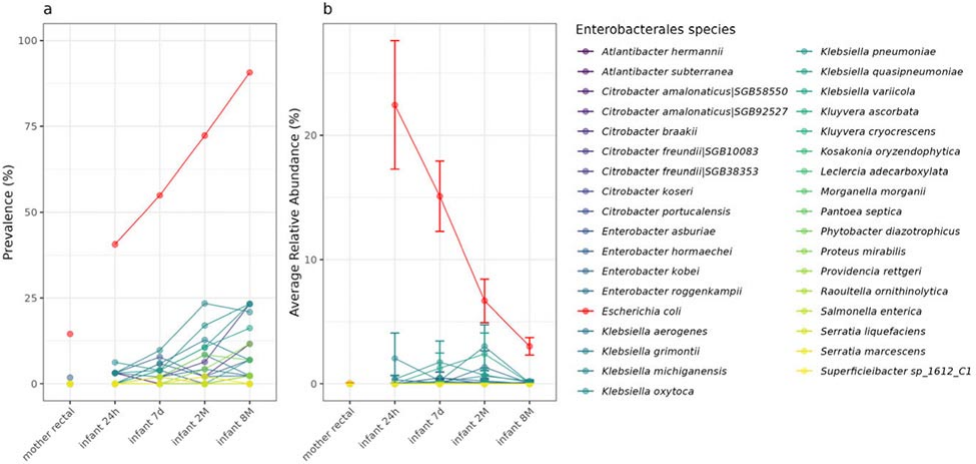
Enterobacterales species prevalence and relative abundance in maternal and infant samples

**Methods:**

Pregnant women were enrolled during labor at a high-volume hospital in Chicago, IL. Exclusion criteria were delivery < 35 weeks gestation, fever, scheduled cesarean, and NICU admission. Maternal rectal swabs were collected prior to delivery, and infant stool was collected at 1-2 days (timepoint1), 7-10 days (t2), 2 months (t3), and 8 months of life (t4). Clinical data were obtained from the medical record and parental survey. Samples were plated on MacConkey + ceftriaxone and underwent shotgun next generation sequencing. Shotgun sequencing results were decontaminated and processed to obtain microbial taxonomic profiling and BL gene identification. CefR culture-based and metagenome-based results were combined to identify each sample’s beta-lactamase status (BL-E negative or positive). Whole genome sequencing was performed for ceftriaxone resistant (CefR) isolates.

**Figure 2.**
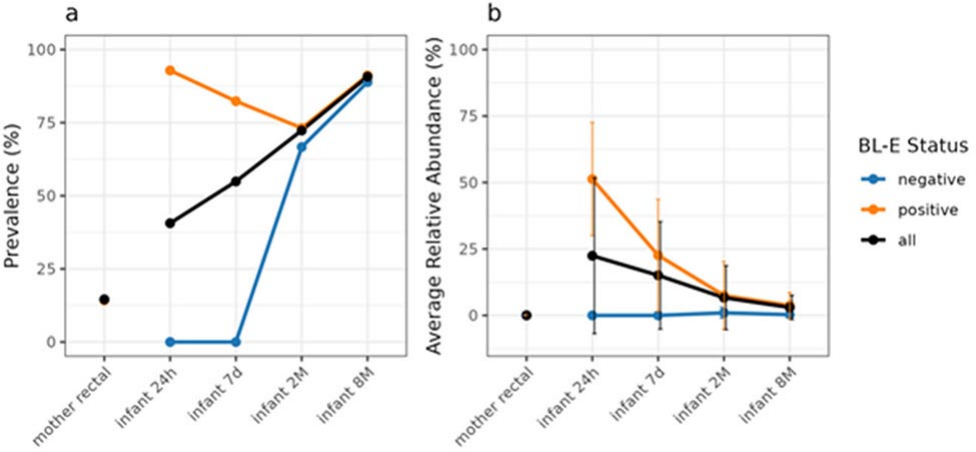
Prevalence and relative abundance of Escherichia coli in mother and infant gut microbiome

**Figure 3.**
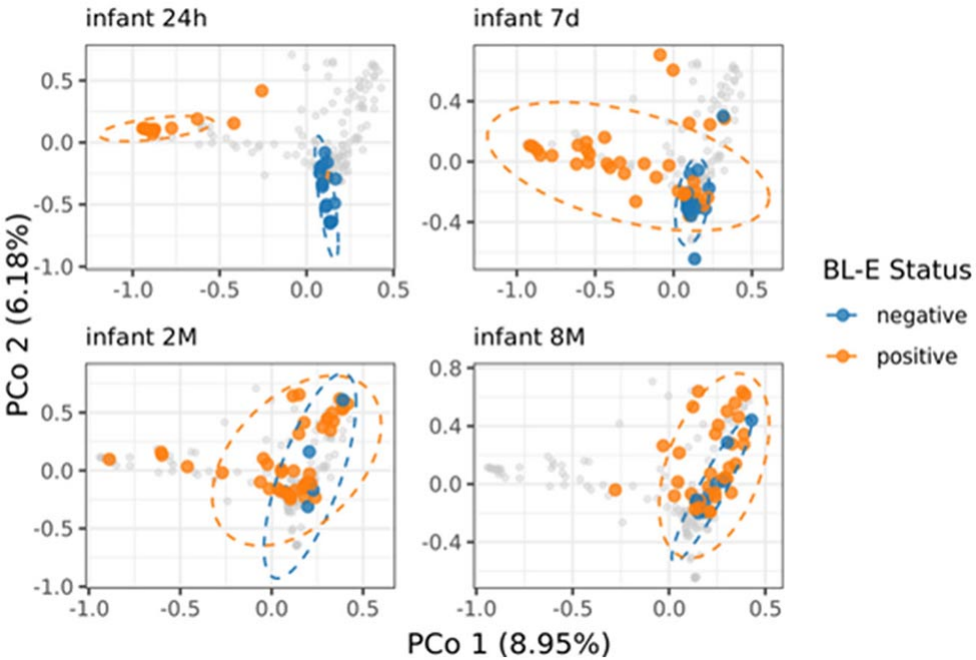
Beta diversity of infant gut microbiome over time grouped by BL-E status combining culture- and metagenome-based identification Kruskal-Wallis test p < 0.01; post hoc pairwise comparisons using Wilcoxon rank sum test p < 0.01 between all timepoints for infant samples

**Results:**

Sixty-one dyads were enrolled 2/2022-1/2023 [Table 1], with 57 parent and 185 infant stool samples (t1=46, t2=50, t3=46, t4=43). CefR-E was identified in 15 parent and 13 infant samples over the study period. Not all infants with CefR-E had CefR-E cultured at subsequent timepoint. Among the 35 Enterobacterales species identified, *Escherichia coli* showed the highest prevalence and relative abundance [Fig 1]. Infant *E. coli* relative abundance decreased over time. Infant *E. coli* prevalence showed an increasing trend over time, with a distinct difference between BL-E positive and negative samples [Fig 2]. Difference in infant microbial community structure and beta diversity between BL-E positive and negative samples was larger for early time points (t1 and t2) compared to later time points (t3 and t4) [Fig 3].

**Conclusion:**

Maternal and perinatal colonization with beta-lactamase resistant Enterobacterales leads to gut microbiome differences in early infancy. The impact of these early life alterations in *E. coli* and diversity warrants further investigation.

**Disclosures:**

All Authors: No reported disclosures

